# Dermoscopy of Bacillary Angiomatosis: Utility in Diagnosis and Therapeutic Control

**DOI:** 10.5826/dpc.1104a88

**Published:** 2021-10-01

**Authors:** Maria Leonor Enei, Francisco Macedo Paschoal, Rodrigo Valdes

**Affiliations:** 1 Brazilian Society of Dermatology. Private Practice, Iquique, Chile; 2 Dermatology Department, Faculdade de Medicina do ABC, Santo André, Brazil; 3 Institute of Histopathology, Histonor, Antofagasta, Chile

**Keywords:** dermoscopy, dermatoscopy, bacillary angiomatosis, HIV, Kaposi sarcoma

## Introduction

Bacillary angiomatosis (BA) is an infectious vascular proliferation caused by *Bartonella henselae* and *Bartonella quintana* Gram-negative bacteria. Its association with HIV was described by Stoler et al in 1983.

The skin lesions begin as small superficial erythematous papules that grow to form friable plaques or nodules surrounded by a flaking collarette. It can compromise internal organs such as the liver or spleen and is considered potentially fatal in immunosuppressed patients, although early treatment leads to complete resolution of the lesions. In patients with HIV, the main differential diagnosis is Kaposi sarcoma (KS), the most prevalent cancer in untreated individuals.

Dermoscopy, a non-invasive diagnostic technique that is useful for the diagnosis of both pigmented and cutaneous vascular lesions, has been used to describe KS lesions not for BA lesions yet. Here we present the dermoscopic description of a case of BA in a patient with no previous HIV diagnosis.

## Clinical Presentation

A 29-year-old male patient consulted the dermatology clinic in January 2020. The patient presented a lesion at the level of the nasal dorsum and covering the beard area. The lesion had a 4-week evolution.

**Figure 1:**
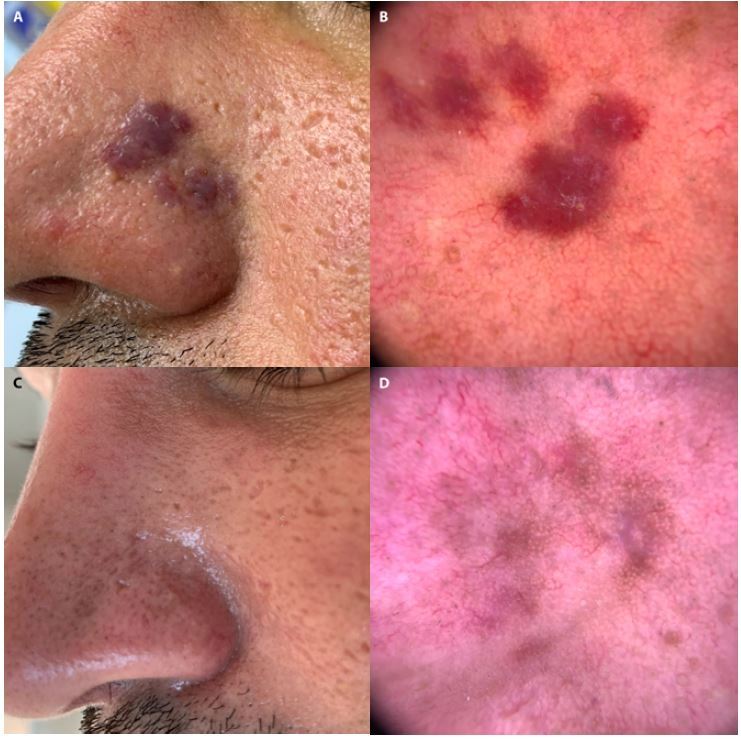
(A) Exam revealed papules and red-purple nodules, grouped and well delimited on the left side of the nose. (B) Dermoscopy showing oval bright red areas and globular structures, with grayish background. Arborizing telangiectasias are present in the periphery. (C) Exam after 3 months of treatment evidenced normal aspect of the skin. (D) Dermoscopy after treatment showed complete disappearance of vascular structures. Only discrete pseudo-red pigment and some arborizing telangiectasias remains.

Clinical examination revealed papules and grouped and well delimited red-purple nodules (Figure 1A). There were no palpable adenopathies or alterations of the oral mucosa. General condition and weight were preserved. Dermoscopic examination (polarized light, DermLite 4) showed oval shapes with bright red areas, and globular structures, with grayish background. Arborizing telangiectasia was seen in the periphery (Figure 1B).

**Figure 2:**
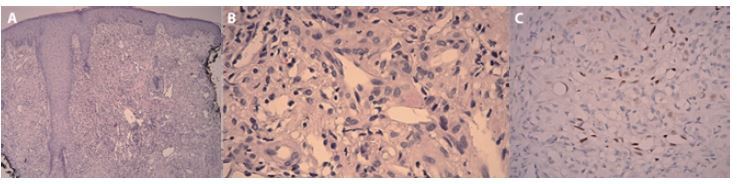
Histopathology. (A) Slight acanthosis and hyperkeratosis in the epidermis. (B) The reticular dermis presents vascular, multifocal, and nodular proliferation composed of small capillary vessels, some with prominent endothelium, extravasation of erythrocytes, infiltration of lymphocytes, and neutrophil polymorphonuclear cells. (C) Immunohistochemical staining (cat scratch disease, Ag B Henselae) revealed isolated bacilliform form**s**.

Upon direct questioning, the patient reported having close contact with house cats. We therefore performed a histopathological study to discriminate between the diagnostic hypothesis of BA versus KS. ELISA test was positive for HIV, with a viral load of 382 IU/mL, CD-4 273/mm3 (424–1509), and lymphocyte count 6,000/uL (4,000 to 10,000). The patient started anti-retroviral treatment (Genvoya^®^) 30 days after. Based on the histopathological and immunohistochemical report (Figure 2), consistent with BA, doxycycline treatment, 200 mg daily, was started. Clinical improvement was evident after 3 months (Figure 1C) as confirmed by control dermoscopy (Figure 1D).

## Conclusions

Here we present the dermoscopic findings of a case of BA in which the vascular component predominated. Upon dermoscopic-histopathological correlation of the clinical presentations, the globular structures, bright red areas, and grayish areas in the background, correspond to the intense proliferation of capillary blood vessels along with the extravasation of red blood cells in the reticular dermis, and a slight acanthosis and hyperkeratosis in the epidermis, respectively.

On the other hand, dermoscopic findings described in KS, namely, the presence of a homogeneous pattern of colours ranging from reddish (46.6%) to blue, pink, whitish (13.3%), or purple [1], is highly suggestive of the diagnosis [2]. The so-called polychromatic structure pattern or “rainbow pattern” has also been highlighted; it is present in all the papular nodular forms of KS and would be expected in our patient [2].

Considering the site of the lesion and telangiectasias in our case, another differential diagnosis that should be considered is that of granuloma faciale. According to larger studies describing granuloma faciale, dermoscopic features should include a marked follicular, and perifollicular white halo, in addition to linear branching vessels [2].

The absence of the above mentioned dermoscopic findings, together with the presence of a marked vascular pattern, directed us to diagnose BA, a disease that is often underdiagnosed.

The complete resolution documented with dermoscopy once again shows the usefulness of this technique for monitoring response to treatments.
